# Psychometric Properties of the Hebrew Modified Dental Anxiety Scale in Adult Israeli Population

**DOI:** 10.3390/ijerph19031499

**Published:** 2022-01-28

**Authors:** Maayan Shacham, Lee Greenblatt-Kimron, Gerry Humphris, Menachem Ben-Ezra, Eitan Mijiritsky

**Affiliations:** 1 School of Social Work, Ariel University, Ariel 40700, Israel; leegr@ariel.ac.il (L.G.-K.); menbe@ariel.ac.il (M.B.-E.); 2 Medical School, University of St Andrews, St Andrews KY16 9AJ, UK; gmh4@st-andrews.ac.uk; 3 Department of Otolaryngology, Head and Neck and Maxillofacial Surgery, Sourasky Medical Center, Tel Aviv 6139001, Israel; mijiritsky@bezeqint.net; 4 The Maurice and Gabriela Goldschleger School of Dental Medicine, Tel Aviv University, Tel Aviv 6997801, Israel

**Keywords:** dental anxiety, anxiety, specific phobia, modified dental anxiety scale, MDAS

## Abstract

Dental anxiety results in the neglect of oral hygiene and poor oral health, requiring an accurate screening tool for dental practitioners to evaluate dental anxiety. The Modified Dental Anxiety Scale (MDAS) is frequently used cross-culturally. The present study aimed to assess the reliability and validity of the Hebrew version of the MDSA. A total of 553 (mean age 35.87 years, SD = 13.14) Israeli participants were recruited through means of social media, mailing lists, and forums. The sample was randomly divided into two population sets. Dental anxiety was evaluated using the Hebrew version of the MDAS. The psychometric evaluation consisted of exploratory factor analysis (study 1, *n* = 274) and confirmatory factor analysis (study 2, *n* = 279). Cronbach’s alpha coefficient was used to assess internal consistency. Results showed high internal consistency (0.93) for the Hebrew version of the MDAS. Confirmatory factor analysis showed a single factor solution. Findings demonstrated 13.4% of Israeli participants with dental anxiety. Younger participants, females, participants with lower education, lower income, and more religious participants reported higher dental anxiety. In conclusion, the Hebrew version of the MDAS demonstrated high reliability and validity. It is recommended to use the Hebrew version of the MDAS to evaluate dental anxiety in Israeli dental settings.

## 1. Introduction

Dental anxiety may be described as an abnormal fear or dread of visiting the dentist, and unwarranted unease during any dental procedures [1]. Dental anxiety, at high intensity, is considered to be a specific phobia, as defined in both the Diagnostic and Statistical Manual of Mental Disorders (DSM-V) [2] and the International Statistical Classification of Diseases and Related Health Problems 11th Revision (ICD-11) [3]. Individuals suffering from elevated levels of dental anxiety are susceptible to a recognizable vicious cycle: their dental anxiety and fear lead to poor attendance at dental practices, which in turn leads to symptom-driven treatment, leading to increased anxiety and fear responsible for avoidance of dental treatment. Dentally anxious patients consequently avoid dental appointments [4], neglect oral hygiene, and demonstrate poor oral health (e.g., high rates of dental caries) [5,6,7,8,9].

In a recent review, Carter (2014) suggests five pathways through which dental anxiety may ensue: Cognitive conditioning, vicarious, verbal threat, informative, and parental. The author (Carter, 2014) also claimed that these pathways may be affected by different etiological factors such as genetic vulnerability, cognitive biases, cognitive content, operant conditioning, preparedness, negative affectivity, and anxiety vulnerability. 

Assessment of dental anxiety can be favorably achieved using self-reported measurements [4]. Dental anxiety questionnaires completed by those who experience psychological difficulties in receiving any dental procedures enable dental care providers to make informed decisions regarding suitable treatment plans, considering their patient’s mental and psychological well-being [10,11,12]. Despite the importance of such questionnaires, most dental care providers tend to trust their own clinical experience when estimating their patients’ levels of dental anxiety, perhaps due to their belief in their capability to ‘diagnose’ a dentally anxious patient or their reluctance to incorporate an additional form to their routine check-ups. Nonetheless, evidence has been reported to support the use of dental anxiety assessments in routine care for patient benefit [13].

Factors that govern the usage of dental anxiety questionnaires in clinical practice and epidemiological studies are: number of questions, complexity, validity, reliability, and reproducibility [14]. Different self-reported questionnaires measure dental anxiety, a popular measurement being the Corah’s Dental Anxiety Scale (CDAS). It is a four-item questionnaire. It has been criticized for omitting the assessment of the respondent’s view to dental anesthesia and for having a complex answering scheme [15]. Studies that have evaluated dental anxiety among the Israeli population have frequently used the CDAS [16,17], showing mixed results regarding the gender effect on dental anxiety levels. Blumer et al. (2018) found that among post-graduate pediatric Israeli staff (instructors and students), females presented higher rates of dental anxiety, while Ratson et al. (2016) found that gender had no significant effect on dental anxiety levels among Israeli adults.

In order to overcome such limitations, a new tool was suggested in 1995, the Modified Dental Anxiety Scale (MDAS) [18] was designed. The MDAS is a five-item questionnaire, with an additional single question regarding local anesthesia, along with uniform responses, thus simplifying the task for the respondent, and assisting the clinician in interpreting item scores. Each item of the MDAS has five consistent responses ranging in increasing order from “not anxious” to “extremely anxious”, with accompanying scores ranging from 1 to 5, corresponding to dental anxiety of increasing severity [18]. The results are then summed together to construct a Likert scale with a minimum score of 5 and a maximum score of 25 [19]. A score of 19 or higher is the cut-off that indicates an individual with elevated risk for dental anxiety [18]. The usage of the MDAS offers various advantages such as being simple, short completion time, and the process of completion does not increase the respondent’s anxiety [20]. The MDAS also has good cross-cultural validity [21], and has been translated into many languages [1,22,23]. 

In summary, there are currently no studies evaluating dental anxiety in the adult Israeli population using the MDAS, as it has yet to be translated to Hebrew and validated. The current study aims to validate the Hebrew version of the MDAS to provide Israeli dental staff an important tool to better estimate their dental patients’ levels of dental anxiety. Providing a dental treatment plan tailored to one’s well-being may aid in easing dental anxiety and eventually improve both the physical (dental) and psychological status of the patient, with additional benefits such as improvements in quality-of-life measures.

In the present study, the psychometric properties of the Hebrew version of the Modified Dental Anxiety Scale are presented. Study 1 aimed to perform exploratory factor analysis, whereas Study 2 aimed to perform confirmatory factor analysis. Both studies were conducted on a convenience sample from the adult Israeli general population.

The overall purpose of this study was to validate the Hebrew version of the MDAS questionnaire. The English version of the MDAS constructed in 1995 [18] and reported a Cronbach’s alpha of 0.93 [18,24]. The MDAS incorporates a Likert rating scale with five possible responses to each question. The questions are related to the respondents’ emotional reactions to a visit to a dental clinic while waiting in its waiting hall, drilling, scaling, and prior to a local anesthesia injection. The responses range from ‘not anxious’ (scoring 1) to ‘extremely anxious’ (scoring 5). A Hebrew language version was produced and back translated by the authors (MS and MB-E). A native English speaker (LK-G) back translated the questionnaire into English and her version was compared with the version produced by the authors. Differences among the versions were resolved by consensus between the authors. In addition, an Israeli dentist and doctor (psychiatric resident) gave independent assessments of the translations.

## 2. Materials and Methods

### 2.1. Sampling and Procedure

We used an internet platform to conduct the survey (Google forms) after gaining approval for the study from the Institutional Review Board of the authors’ (MS, LG-K, MB-E) university. Inclusion criteria included Israeli adults older than 18 years of age, with exclusion criteria being younger than 18 years of age. During the period of 13 November to 7 December 2019, participants were approached using social media (Facebook, WhatsApp), dedicated mailing lists (MS), and forums (*n* = 553). The mean age of these participants was 35.87 years (SD = 13.14, range = 18–76), 62.9% were female (*n* = 348), 69.6% (*n* = 385) were in a committed relationship, 58.2% (*n* = 322) were secular in their degree of Judaism spirituality, and mean years of education was 14.99 (SD = 3.09). Each participant signed an electronic informed consent form. The sampling and procedure in Study 2 was identical to Study 1.

### 2.2. Measures

A basic socio-demographic questionnaire was used for the following: age (coded in years), gender (coded as ‘1 = male, 2 = female’), marital status (coded as ‘1 = not being in a committed relationship, 2 = being in a committed relationship’), years of education (coded in years), degree of Judaism spirituality (coded as ‘1 = secular, 2 = traditional, 3 = religious, 4 = ultra-orthodox’), and level of income relative to the average salary in Israel (coded as ‘1= highly below average, 2 = lower than average, 3 = average, 4 = higher than average, 5 = much higher than average’). In addition, as depicted previously, the Hebrew MDAS scale items were utilized. The measures in Study 2 were identical to study 1.

### 2.3. Statistical Analysis

Descriptive statistical analysis was conducted for sociodemographic factors. In addition, an exploratory factor analysis (EFA) was conducted (principal component analysis) using Horn’s Parallel Analysis (*paran* procedure) including the 5 MDAS items (items were rated on a 5-point scale ranging from 1 = ‘not anxious’, to 5 = ‘extremely anxious’), thereby producing a basic component matrix followed by an orthogonal (varimax) rotation method if more than a single factor indicated. Confirmatory factor analysis was run (*sem* procedure) with the conventional methodology of setting a single item to unitary loading to ‘scale’ the measure. Standardized solutions were requested and modification indices were examined for evidence of correlated residual errors. The total sample was randomly divided into two separate sub-samples for EFA (study 1, *n* = 274) and confirmatory factor analysis (CFA, study 2, *n* = 279). Data were analyzed utilizing STATA 15 (Stata CORP, College Station, TX, USA). Alpha level was 0.05 (2 sided) for all tests. The sample was divided into two separate sub-samples in order to better evaluate factor analyses. If both EFA and CFA were to be conducted on the same total sample, its results would be difficult to interpret as very similar results would be obtained. Thus, in order better interpret when conducting factor analyses, the total sample was divided.

The purpose of Study 2 was to conduct the confirmatory factor analysis (CFA) of the MDAS in the second sub-sample (*n* = 279). We performed a confirmatory factor analysis (CFA). The analysis was conducted using STATA 15 (Stata CORP, College Station, TX, USA). Overall goodness of fit for the analysis of the proposed unidimensional measurement model was assessed by a variety of indices: Tucker–Lewis index (TLI, recommended result should be higher than 0.95); comparative fit index (CFI, recommended result should be higher than 0.95); Standardized root mean square (SRMR, cut-off value 0.08 or below); Coefficient of determination (CD, ranged between 0 and 1 with closer to 1 suggesting better model fit); and the root mean square error of approximation (RMSEA, recommended result should be lower than 0.1) (for more information about the goodness of fit cutoffs, see [25,26,27,28,29,30]). Following the CFA, we conducted a correlation matrix for all the measures used in each sample. Beyond that, we conducted a regression analysis to learn of the association between demographic variables and dental anxiety. The demographic factors were age, sex, relationship status, education, income, and religiosity. The dependent variable was dental anxiety. An a priori test revealed no multicollinearity. The tolerance ranged from 0.634 to 0.905 and the Variation Inflation Factor (VIF) ranged from 1.105 to 1.577.

## 3. Results

### 3.1. Exploratory Factor Analysis—Component Matrix

Descriptive statistics about basic sociodemographic factors are presented in Table 1.

The cut-off for elevated risk for dental anxiety in the MDAS is 19 and above. In the current study, 74 participants out of 553 showed elevated risk for dental anxiety resulting in 13.4% of the sample.

The results of the linear regression showed that the model was significant. The R was 0.343 and the R^2^ was 0.118 (F change = 12.141; DF = 6545; *p* < 0.0001). The results revealed the dental anxiety was associated with younger age (β = −0.118; t = −2.563; *p* = 0.011), being a female (β = 0.204; t = 4.825; *p* < 0.001), and being more religious (β = 0.122; t = −2.829; *p* = 0.005). Relationship status, education, and income were not found to be associated with dental anxiety.

**Figure 1:**
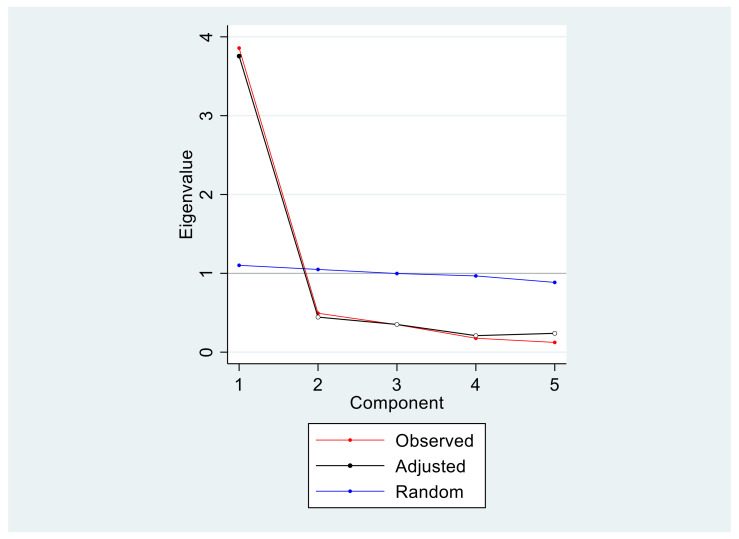
Scree plot (Study 1). As can be noted, one-component solution was found for the Hebrew version of the MDAS. The observed (red) and adjusted (black) both illustrate and support the one-component solution.

The exploratory factor analysis result revealed only one factor. In order to ensure that we did not have more than one concept underlying the Hebrew version of the MDAS, the exploratory factor analysis of the five-item MDAS found only one eigenvalue was greater than 1.00: 3.82 (explained variance = 78%), with a smooth continuous drop for the remaining eigenvalues. This feature made the clearest break in the Scree Plot between the first and second factor, again highly suggestive of a one-factor solution. See Table 2 and Figure 1 for further information.

### 3.2. Confirmatory Factor Analysis (CFA)

The results of the CFA showed that a one-factor solution showed a good fit. This solution incorporated a single correlated error into the model as indicated by a high modification index for this covariance parameter estimate (>10). See Table 3 for further information. The Cronbach’s alpha for the MDAS in the current study was 0.93.

## 4. Discussion

Dental anxiety can result in the negligence of oral hygiene and poor oral health [5,6,7,8]. An accurate screening tool is advocated that will easily meet the needs of dental care providers to make informed decisions regarding suitable treatment plans with patients with dental anxiety. The MDAS has many advantages for such screening [21], has good cross-cultural validity [22], and has been translated into many languages [1,23,24]. Nevertheless, there is no translated and validated Hebrew version to date. The present study, therefore, examined the psychometric properties of the Hebrew version of the MDAS. The first study aimed to determine the dimensionality of validate the Hebrew version of the MDAS questionnaire, while the second study performed confirmatory factor analysis.

A factor analysis of demonstrated singular dimensionality of the Hebrew version of the MDAS. Results revealed that Cronbach’s alpha coefficient for the Hebrew version of the MDAS was high (0.925–95% CI’s 0.905–0.941), which is similar to the Cronbach’s alpha value of the Italian (0.92; [31]) and Greek (0.9; [32]) versions, and the original English version of the United Kingdom (0.9; [18]). Moreover, Cronbach’s alpha value of the Hebrew version was higher than the Nepali (0.78; [33]), Indian (0.78; [34]), Arabic (0.87; [22], Spanish (0.88; [35]), and Japanese (0.88; [36]) versions. The results clearly demonstrated a unidimensional solution.

The Confirmatory Factor Analysis showed a single factor solution. Of interest was the inclusion of a single correlated error introduced into the model between items one and two. These two items have been identified previously as a subscale of anticipatory dental anxiety [37]. The remaining three items refer to specific dental procedures. Hence, the researcher may consider this as a subscale of dental treatment anxiety. Investigators are recommended to report the total score but use the two subscales as exploratory for detailed analysis.

In the present study, 13.4% percent of participants reported phobic levels of dental anxiety according to cut off scores. The prevalence of dental anxiety in the current study, i.e., in the Israeli population, is higher than studies conducted among populations in Nepal [33], Japan [36], India [38], Finland [21], and Malaysia [1], yet lower than those in Northern Ireland [21] and Italy [32]. It was recently noted that this diversity may stem from variations in sampling methods across populations [36]. The present study demonstrated that age was inversely linked with dental anxiety, namely, younger participants had higher MDAS scores. This finding supports some studies [19,34,38], while others found higher dental anxiety among older participants [39] or no differences in MDSA scores by age [40,41]. In the current study females reported higher levels of dental anxiety than males, which is consistent with findings in samples in the UK [42], Japan [36], Italy [31], Greece [32], Saudi Arabia [43], Malaysia [1], Turkey [39,44], and China [23]. However, sex differences were not reported in the Nepali sample [33]. A negative correlation was found between dental anxiety and education in the present study, namely, those with lower education reported higher dental anxiety. This finding corresponds with previous studies [34,38,41]; furthermore, a negative link was found between lower income with dental anxiety, namely, those with lower income reported higher dental anxiety. This finding supports those of previous studies [1,5,45,46]; nevertheless, is it contrary to a study demonstrating a positive correlation between dental anxiety and higher income [47], as well as studies that demonstrated no differences in dental anxiety and participants’ monthly income [38,46,48]. Finally, in the present study a correlation was found between the participants’ level of religiosity and their level of dental anxiety, namely, those who were more religious reported higher dental anxiety.

The present study has some limitations; therefore, findings should be interpreted cautiously. First, the study was based on self-report questionnaires; therefore, recall bias may affect the results. Second, participants were recruited via web-based platforms; as a result, the study sample may be biased towards those who are active on these networks.

## 5. Conclusions

The Hebrew version of the MDAS demonstrated high reliability and some evidence for validity. Therefore, it is recommended that the Hebrew version of the MDSA be used to quantify dental anxiety in the Israeli population. This is essential as the Hebrew version of the MDAS is likely to provide Israeli dental staff a vital tool for a more accurate assessment of their dental patients’ levels of dental anxiety.

## Figures and Tables

**Table 1:** Descriptive presentation about basic sociodemographic data among current study participants (*n* = 553).

Sociodemographic Characteristic	N, %	Mean (±SD), Range
**Sex**		
Female	348, 62.9%	
Male	205, 37.1%	
**Age (years)**		
		35.87 (±13.14), 18–76
**Years of education**		
		14.99 (±3.09), 2–27
**Household income**		
Highly below average	134, 24.2%	
Lower than average	110, 19.9%	
Average	120, 21.7%	
Higher than average	139, 25.1%	
Much higher than average	50, 9%	
**Marital status**		
Not in committed relationship	168, 30.4%	
In a committed relationship	385, 69.6%	
**Degree of religiosity**		
Secular	322, 58.2%	
Traditional	99, 17.9%	
Religious	92, 16.6%	
Ultra-orthodox	40, 7.2%	

**Table 2:** Exploratory Factor Analyses of the Hebrew Version of MDAS Factor Loadings (Study 1).

	Base Matrix
MDAS items	Factor 1
1	0.883
2	0.901
3	0.878
4	0.774
5	0.787

**Table 3:** Model Evaluation Overall Fit Measurement for the Hebrew Version of MDAS, data analyzed with STATA 15.

Fit Measurement Fit Indices	Recommended Value	(*n* = 279)
Maximum Likelihood (standardized regression weight)		
X^2^ (5)	N/A	13.15
*p* > X^2^	N/A	0.01
Df	N/A	1
SRMR	≤0.08	0.02
CD	N/A	0.93
TLI	≥0.95	0.98
CFI	≥0.95	0.99
RMSEA	≤0.1	0.091
